# Anesthetic management of cesarean hysterectomy using intra-aortic balloon occlusion in a patient with Fontan circulation and placenta increta: a case report

**DOI:** 10.1186/s40981-023-00611-1

**Published:** 2023-04-24

**Authors:** Eriko Ohsugi, Rie Kato, Yuki Hosokawa, Katsunori Oe

**Affiliations:** Department of Anesthesiology, Showa University School of Medicine, 1-5-8 Hatanodai, Shinagawa-Ku, Tokyo, 142-8555 Japan

**Keywords:** Fontan circulation, Intra-aortic balloon occlusion, Obstetric hemorrhage, Placenta accreta spectrum, Arterial pressure-based stroke volume

## Abstract

**Background:**

In patients with Fontan circulation, hemorrhage can cause life-threatening circulatory collapse, since Fontan circulation strongly depends on the preload. Furthermore, parturients with placenta accreta spectrum are at a high risk of rapid and massive hemorrhage. Herein, we report the case of an intra-aortic balloon occlusion used for a Fontan-palliated parturient with placenta increta with successful anesthetic management.

**Case presentation:**

A 35-year-old-female with Fontan circulation diagnosed with placenta increta underwent a cesarean hysterectomy. The main goal during anesthetic management was to maintain sufficient preload. Infrarenal intra-aortic balloon occlusion was used to reduce intraoperative hemorrhage. The hemodynamic changes caused were well tolerated in this case.

**Conclusions:**

Intra-aortic balloon occlusion was used in a Fontan-palliated parturient with placenta increta with successful anesthetic management.

## Background

Long-term survival rate in patients with congenital heart disease has improved; consequently, more patients with Fontan circulation are reaching child-bearing age.^1^Fontan palliation establishes single-ventricle circulation by directing venous blood from the body to the pulmonary artery bypassing the ventricle. Cardiac output in such patients is highly dependent on the preload; thus, they are susceptible to hypotension due to blood loss [1, 2]. Patients with placenta accreta spectrum (PAS) are at a high risk of rapid and massive peripartum hemorrhage [3]. This can predispose parturients, especially those with Fontan palliation, to life-threatening circulatory collapse. We report the case of an intra-aortic balloon occlusion (IABO) used for a Fontan-palliated parturient with placenta increta with successful anesthetic management.

## Case presentation

**Fig. 1:**
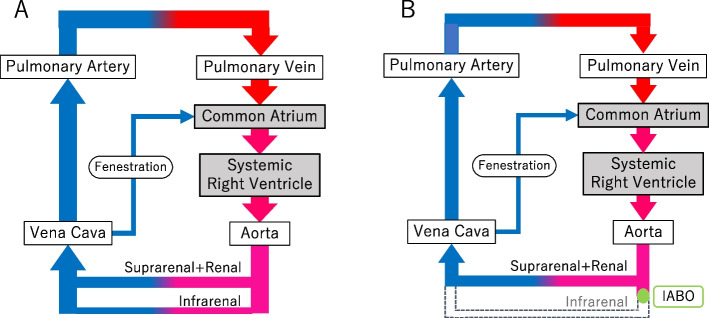
Circulatory flow in the current Fontan-palliated patient **A** without infrarenal IABO and **B** with IABO. **A** Venous blood flows back directly to the pulmonary artery without passing through the atrium and ventricle. **B** When infrarenal IABO is applied, cardiac output is assumed to be reduced. IABO, intra-aortic balloon occlusion

A 35-year-old woman with gravidity 2 and parity 1 was referred to our hospital at 21 weeks of gestation because of Fontan circulation. This was a suspected PAS case with a low-lying placenta that overrode the previous cesarean uterine scar. The patient underwent Blalock-Taussig shunt surgery at 0 and 2 years of age because of a single right ventricle and pulmonary atresia. She also underwent Fontan palliation at age 7 (Fig. 1A). Her previous cesarean delivery under general anesthesia for preterm labor with breech positioning at 28 weeks of gestation was complicated by post-operative pulmonary edema requiring a prolonged hospital stay. After her first pregnancy, she maintained a baseline of New York Heart Association Functional Classification I. Echocardiography findings remained stable, with almost normal right ventricle contractility and mild atrioventricular valve regurgitation. Her hemoglobin and brain natriuretic peptide levels (BNP) were 15 − 16 g/dl and 20 − 35 pg/ml, respectively.

Her height and weight at presentation were 154 cm and 49 kg, respectively. Her blood pressure, heart rate, and percutaneous arterial oxygen saturation were 89/56 mmHg, 60 beats/min, and 91% (room air), respectively. She was on carvedilol 1.25 mg/day and not on thromboprophylaxis due to a history of subchorionic hematoma. Echocardiography showed a right ventricular fractional area change of 38% and mild aortic and atrioventricular regurgitation. The diameter of the inferior vena cava was 10 mm, and fenestration flow was 1.5 m/s. Her hemoglobin level was 12.0 g/dl, and BNP was 22 pg/ml. A cesarean hysterectomy with infrarenal IABO under general anesthesia was planned. Both neuraxial anesthesia followed by conversion to general anesthesia at hemorrhage and general anesthesia were considered. The anesthetic method was chosen in accordance with the patient’s preference and the ability to use transesophageal echocardiography (TEE).

She presented with 430 ml of vaginal bleeding at 26 6/7 weeks gestational age. Red blood cells (280 ml) were transfused, and an emergency cesarean hysterectomy was performed. Standard monitors were placed in the operating room. An arterial line was inserted into the left radial artery, and arterial pressure-based cardiac index (apCI), stroke volume (apSV), and stroke volume variation (SVV) were monitored (FloTrac Sensor™; Edwards Lifesciences, Tokyo). Remifentanil 0.1 μg/kg/min, midazolam 5 mg, and fentanyl 150 μg were administered in titration, and tracheal intubation was performed with rocuronium 50 mg. Two 18-gauge peripheral venous lines were placed. A central venous catheter was placed via the right internal jugular vein, and the central venous pressure (CVP) was monitored. An intra-aortic balloon tip was placed distal to the renal artery. Anesthesia was maintained with 1.5% sevoflurane, continuous remifentanil 0.2 − 0.3 μg/kg/min, and intermittent boluses of fentanyl and rocuronium during the surgery. The inspiratory oxygen concentration was 67% throughout the surgery.

**Fig. 2:**
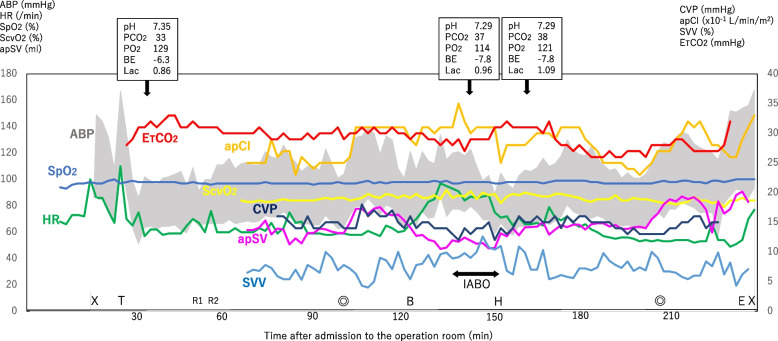
Trends in hemodynamic parameters during anesthesia. Minute ventilation is constant during surgery. Further, FiO_2_ is maintained at 0.67 throughout anesthesia, and abdominal pressure is not applied at delivery of the baby. Oxytocin is not administered after delivery. HR, heart rate, SpO_2_, percutaneous arterial oxygen saturation, ABP, arterial blood pressure, ETCO_2_, end-tidal carbon dioxide partial pressure, ScvO_2_, central venous oxygen saturation, apCI, arterial pressure-based cardiac index, CVP, central venous pressure, SVV, stroke volume variation, apSV, arterial pressure-based stroke volume. X, anesthesia start/finish, ◎, operation start/finish, B, baby delivery, T, intubation, E, extubation, IABO, intra-aortic balloon occlusion, H, hysterectomy, R1, insertion of the IABO sheath, R2, insertion of the transesophageal echocardiography probe

Before delivery, apSV, SVV, and CVP were approximately 70 ml, 5 − 10%, and 15 mmHg, respectively. ApSV started to decrease immediately post-delivery (Fig. 2), and a rapid infusion of crystalloids was initiated. Transvaginal bleeding was noted 5 min after the delivery, and a rapid blood transfusion was thus administered. Since apSV further decreased to less than 50 ml, suggesting preload depletion, IABO was initiated, and the bleeding decreased instantly. Neither apSV nor CVP fell, irrespective of the increase in SVV, suggesting that the preload was not reduced during IABO. After the uterus was excised, the balloon was deflated. The IABO duration was 15 min.

The total blood loss was 2000 ml; accordingly, 1400 ml of crystalloids, 840 ml of red blood cells, and 480 ml of fresh frozen plasma were infused. Oxytocin was not administered during the surgery. The Apgar scores of the neonate at 1/5 min were 1/5, respectively. The child was intubated and transferred to the neonatal intensive care unit. The mother was extubated and transferred to the intensive care unit. On post-operative day 5, she developed pulmonary edema, with a rise in BNP to 482 pg/ml, which improved with furosemide. She was discharged on post-operative day 17. Further, pathology confirmed the diagnosis of placenta increta.

## Discussion

Dynamic physiological changes at delivery make anesthetic management very challenging in Fontan-palliated parturients [2]. The principles of hemodynamic management in Fontan circulation include maintaining preload and cardiac contractility and avoiding an increase in pulmonary vascular resistance [1, 2]. Successful anesthetic management of cesarean delivery in patients with Fontan circulation has been reported [4, 5]. However, this is the first report of a Fontan-palliated parturient complicated with PAS. The perinatal cardiac risk in this patient was relatively low for a Fontan-palliated parturient. However, rapid and massive hemorrhage is a known complication in patients with PAS [3]. Therefore, in this case, the primary goal of anesthetic management was to maintain a sufficient preload.

In this case, apSV reflected the changing preload during rapid hemodynamic fluctuations, such as hemorrhage and IABO (Fig. 2). Notably, apSV is arterial pressure-based cardiac output (apCO)/heart rate. Further, apCO allows accurate cardiac output measurement in normo- and hypo-dynamic states without strong changes in the vascular tone [6]. This case could be categorized by such a hemodynamic state based on the observation that both peripheral vascular resistance, as calculated from the mean arterial pressure, CVP, and apCO, and ventricular contractility, as assessed using TEE, remained within normal ranges during the surgery.

Although CVP, SVV [6], and ventricular size are considered to be good indicators of preload in the general population, there are no standard indices for assessing the preload in Fontan-palliated patients for non-cardiac surgery. A previous study has reported CVP as a useful index of circulatory blood volume in a Fontan-palliated patient during neurosurgery [7]. Further, ventricular filling assessed using TEE was a good indicator of preload in spinal surgery [8]. However, the credibility of SVV in Fontan-palliated patients is yet to be determined [7, 9, 10]. In the current case, both SVV and ventricular size were unreliable as preload indicators since SVV fluctuations were considerably strong throughout anesthesia. Further, the ventricular size remained constant during rapid bleeding, and apSV was a reliable preload indicator in this case. Measuring apSV is easier and less invasive than CVP or TEE, making it a convenient parameter. However, more Fontan-palliated cases are needed to confirm its reliability as an alternative to CVP or ventricular size.

The use of IABO decreases blood loss during delivery in patients with PAS [11, 12]. However, its use in Fontan-palliated patients could impose adverse hemodynamic effects. Infrarenal aortic cross-clamping, which mimics infrarenal IABO, increased systemic vascular resistance by 30 − 50%. It also reduced right ventricular volume by 10 − 13%, suggesting a decreased preload. Consequently, aortic cross-clamping reduced the cardiac index by 23 − 38% [13, 14]. In the present case, the patient was anticipated to tolerate the transient increase in systemic vascular resistance because her ventricle was not failing. However, it was a matter of concern whether the preload reduction by infrarenal IABO (Fig. 1B) would result in an extreme decrease in cardiac output since Fontan circulation strongly depends on preload. Another minor concern was the elevation of pulmonary vascular resistance due to metabolic acidosis following balloon deflation [15]. Consequently, it was planned that IABO would not be used prophylactically, rather only when the bleeding was excessive and when maintaining preload would be difficult. Contrarily, neither the arterial pressure, apSV, apCI, nor ETCO_2_ was reduced, indicating that the cardiac index was maintained. Rapid blood transfusion (900 ml) may have contributed to maintaining the cardiac index. Upon completion of IABO, the balloon was deflated by half for 3 min before complete deflation. Moreover, CVP remained below the pre-delivery value, and fenestration flow did not decrease, suggesting no signs of pulmonary hypertension. A stable pulmonary vascular resistance at deflation may be due to the gradual deflation of the balloon and the short duration of infrarenal IABO, 15 min. Further, an increase in apSV and CVP after balloon deflation may have been caused by the return of infrarenal circulation, which did not occur during IABO (Fig. 1B). The adverse hemodynamic effects of IABO were not significant. Further, the blood loss was approximately 1800 ml 30 min after hysterotomy. Notably, IABO could have been started immediately after the delivery of the fetus to reduce the hemorrhage. Prophylactic use of IABO can be considered in Fontan-palliated patients in the future.

In summary, this case reports on the anesthetic management of a parturient with Fontan circulation who underwent a cesarean hysterectomy for placenta increta. The adverse hemodynamic effects of IABO were not significant. When the ventricular contractility and peripheral vascular resistance are adequate, arterial pressure-based SV can be a good surrogate for preload in Fontan-palliated patients.

## Data Availability

Data sharing is not applicable to this article as no datasets were generated or analyzed during the current study.

## Abbreviations

PAS: Placenta accreta spectrum
IABO: Intra-aortic balloon occlusion
BNP: Brain natriuretic peptide level
TEE: Transesophageal echocardiography
apCI: Arterial pressure-based cardiac index
apSV: Arterial pressure-based stroke volume
SVV: Stroke volume variation
CVP: Central venous pressure
apCO: Arterial pressure-based cardiac output
ETCO_2_: End-tidal carbon dioxide partial pressure
